# Body Mass Index and Comorbidities in Adult Severe Asthmatics

**DOI:** 10.1155/2014/607192

**Published:** 2014-05-28

**Authors:** Andreina Bruno, Elisabetta Pace, Fabio Cibella, Pascal Chanez

**Affiliations:** ^1^Istituto di Biomedicina e Immunologia Molecolare (IBIM), Consiglio Nazionale delle Ricerche (CNR), Via Ugo La Malfa 153, 90146 Palermo, Italy; ^2^Département de Pneumoallergology, AP-HM, Laboratoire d'Immunologie, Inserm CNRS U 1067, UMR7333, Aix Marseille Université, 13009 Marseille, France

## Abstract

Both severe asthma and obesity are growing health problems. Severe asthma leads to a poor quality of life. The relationship among BMI, comorbidities, and severe asthma control in adults is still unclear. The aim of the study is to better understand the effect of the comorbidities as atopy, type II diabetes, OSAS, gastroesophageal reflux, hypertension, cardiovascular diseases, osteoporosis, infections, and psychological factors with BMI on asthma control in a cohort of adult severe asthmatics. One hundred and two patients were enrolled in a cross-sectional study assessing asthma control, treatments, pulmonary function, inflammatory markers, and comorbidities. Patients were divided into 3 classes according to BMI: normal weight, overweight, and obese. We found that the optimal state of asthma control is lower. whereas the score of Asthma Control Questionnaire, the number of asthma exacerbations during last year, the oral corticosteroids requirement during the previous year, and the LABA treatments are higher in obese than in overweight and normal weight severe asthmatics. The number of subjects with type II diabetes and OSAS are higher among obese and overweight patients than in normal weight asthmatics. In conclusion, BMI represents *per se* a factor for the deterioration in disease control in severe asthma.

## 1. Introduction

Asthma and obesity are two important and growing population health issues in industrialized countries. A recent report indicates that an estimated 25.7 million people in the United States had asthma in 2010 [1]. On the other hand, the prevalence of obesity is very high worldwide and it represents a health and economic burden for each country. The World Health Organization has reported that obesity has dramatically increased during the last few decades. In 2009-2010, more than one-third of US adults (35.7%) were obese [2, 3], not only in United States but also in some Western and developing countries [4]. In this scenario, an estimated 300,000 deaths per year are directly attributable to obesity, mainly due to heart diseases, diabetes, cancer, asthma, obstructive sleep apnea syndrome (OSAS), arthritis, and psychological disturbances, leading to the concept that obesity represents a risk factor for several pathologies in different clinical conditions [5]. Particularly, overweight and obesity are associated with a dose dependent increase of the risk of having asthma [6] and obesity appears to be a predisposing factor for the asthma onset, both in adults and in children, as assessed by several cross-sectional studies [7]. Obesity could make asthma more difficult to control and to treat, whereas weight-loss studies have shown substantial improvements in the clinical status, lung function, symptoms, and asthma control in severe asthmatics with high body mass index (BMI) and are associated with a universal improvement of asthma management in the obese patient [8, 9]. However, the mechanism linking obesity and asthma is still a controversial issue. Another major issue is to understand whether asthma symptoms in obese patients are often so difficult to control mainly due to comorbid factors. The definition of severe asthma is based on a combination of symptoms, inflammatory markers, the degree of bronchial obstruction, the intensity of treatment, and dose of inhaled corticosteroids necessary to obtain good control of asthma [9]. Control of asthma is defined according to three health states which were qualified as optimal, suboptimal, and unacceptable control (states 1, 2, and 3) and the concept of control is relevant only if it leads to a therapeutic decision [10, 11]. It has been assessed that some comorbidities are associated with frequent exacerbations in severe asthma, including gastroesophageal reflux, psychological dysfunction, OSAS, and obesity [12]. Based on these evidences in this study, we investigated the potential role of BMI on severe asthma control using an enrolled cohort of asthmatics, taking into account triggers and precipitating factors such as metabolic disorders, cardiovascular diseases, infections, and psychological factors.

## 2. Material and Methods

### 2.1. Study Design

This cross-sectional study was performed on asthmatic outpatients affected by severe asthma. All asthmatics were nonsmokers, known by a chest specialist at least for six months, and were selected according to the American Thoracic Society criteria [13]. Severe asthma and exacerbation rate during the last year were defined according to the GINA guidelines [14, 15]. Severe asthma was defined according to the following clinical features: daily symptoms, frequent exacerbation, frequent nocturnal asthma symptoms, limitation of physical activities, FEV_1_ or PEF ≤ 60% predicted, and PEF or FEV_1_ variability > 30%. Asthma exacerbation was defined as an unscheduled visit to a health care provider or a need for a given patient to increase the oral corticosteroid dose. During a three-month recruitment period, all consecutive asthmatic subjects attending the asthma clinic of a public hospital and fulfilling the inclusion criteria were required to participate in the study. All patients performed spirometry, skin prick test, total IgE, and blood eosinophil levels assessments. The following comorbidities were investigated and treated according to the best medical state of the art: type II diabetes, OSAS, gastroesophageal reflux, systemic hypertension, left ventricle failure, osteoporosis, bronchiectasis, tuberculosis in the past, and psychological factors. The study fulfilled the criteria of the reference Ethic Committee and was in agreement with the Helsinki Declaration. All subjects had given their written informed consent.

### 2.2. Subjects

At the beginning of the study, clinical characteristics and detailed history of the disease were obtained using a previously validated questionnaire [15]. In particular, information about occupation, work disability, and sputum production was collected. The changes in symptoms, the *β*2 agonist use, the lung function parameters, and composite scores were considered to define asthma control. A validated questionnaire, asthma control questionnaire (ACQ, French version for France), was used to collect the composite scores and for assessment of the severity of asthma as previously described [16]. The ACQ is a validated 7-item questionnaire that asks patients to recall their experiences during the previous week and respond to each question on a 7-point scale, which ranges from 0 (well controlled) to 6 (extremely poorly controlled). For each subject, the mean score for the 7 questions was then calculated. The control of asthma was defined according to three health states which were qualified as optimal, suboptimal, and unacceptable control as previously described [10]. Patients were excluded if having any potential confounding diagnosis. They were also excluded if they presented with any persistent environmental trigger, COPD, or other differential diagnosis according to our current clinical assessment. Patients were divided into 3 classes related to their BMI (expressed as kg/m^2^) following the clinical guidelines of an expert panel on the identification, evaluation, and treatment of overweight and obesity in adults [17]: normal weight, 18.5 < BMI < 25.0; overweight, 25.0 ≤ BMI < 30.0; obese, BMI ≥ 30.

### 2.3. Pulmonary Function Tests and Skin Prick Test

Spirometric measurements, including forced expiratory volume in the 1st second (FEV_1_) and forced vital capacity (FVC), were recorded (SensorMedics, mod. *V*_max⁡_ series 229). The pulmonary function results were expressed as percentage of predicted normal values (% predicted) following predicted values from Quanjer et al. [18]. IgE-mediated allergy to inhalants and foods was studied using skin allergy prick tests.

### 2.4. Blood Processing

An aliquot of blood samples was used to test serum total IgE (Phadebas CAP System, Pharmacia Diagnostics AB, Uppsala, Sweden) and for total and differential cell count. Blood eosinophil count was estimated (cells/mm^3^).

### 2.5. Statistical Analysis

Means and standard deviations or medians and interquartile ranges were calculated for continuous data (for normally and not normally distributed variables, resp.) and percentages for categorical data. Differences in frequency distribution of categorical variables were evaluated by Chi-square test. One-way analysis of variance and Kruskal-Wallis tests were used for comparison of normal and not normal variables, respectively, among subgroups. Normality of variable distributions was evaluated by Kolmogorov-Smirnov test. All computations were performed by StatView statistical software package (SAS Institute, Cary, NC, USA). A *P* value <0.05 was considered statistically significant.

## 3. Results and Discussion

### 3.1. Subjects

One hundred and two adult severe asthmatic patients (45 M, 57 F) were included in the study. The mean age was 56.6 years (±14.0 SD). The normal weight, overweight, and obese severe asthmatics were 42, 36, and 24, respectively. A complete description of the demographic and clinical characteristics of the patients is reported in Table 1.

### 3.2. Pulmonary Function Tests, Asthma Control and Exacerbations

Mean FEV_1_, FVC, and FEV_1_/FVC did not significantly differ among studied subgroups at baseline level (Table 1). Among obese subjects, the optimal state of asthma control was significantly lower than in overweight and in normal weight severe asthmatics (*P* = 0.023), whereas the ACQ score and the number of asthma exacerbation episodes during the last year were significantly higher or borderline (*P* = 0.005 and *P* = 0.052, resp.) (Table 1). The duration of asthma, the hospitalization events, the intensive care unit events, and the oral corticosteroids requirement ever in life were similar in the three categories studied (Table 1).

### 3.3. Pharmacological Treatment

The oral corticosteroids requirement during the previous year and the long acting beta2-agonists (LABA) treatment were significantly higher (*P* = 0.029 and *P* = 0.027, resp.) in obese than in overweight and in normal weight severe asthmatics (Table 1). On the other hand, neither aspirin intolerance nor antidepressant and antihistaminic treatments were different among the three categories studied (Table 1).

### 3.4. Inflammatory Markers and Comorbidities

In all the studied groups, neither eosinophils numbers nor atopy or IgE levels were associated with BMI and asthma severity (Table 2). Furthermore, in Table 3 we reported the comorbidities studied for the association with BMI: we found that at least one comorbidity (*P* = 0.031), the type II diabetes (*P* < 0.0001), and OSAS (*P* = 0.0081) were significantly more frequent in obese and in overweight than in normal weight severe asthmatics. The other comorbidities evaluated were not significantly different among the three categories studied (Table 3).

## 4. Discussion 

A large amount of the literature indicate an increased relative risk of asthma in overweight and obese patients, but currently this issue is still controversial and the mechanistic basis underlying the association between obesity and asthma has not been established. For instance, a recent meta-analysis assesses that overweight and obesity are associated with higher incidence of multiple comorbidities including type II diabetes and asthma [19]. In fact, more recently, it has been demonstrated that obesity and diabetes are the components of metabolic syndrome driving the increase of asthma prevalence in adults [20]. Despite the fact that the abundant literature has been produced on this topic, the ultimate cause of the relationship between high BMI and severe asthma in adults has not been identified and the influence of obesity with other comorbidities on asthma control is a matter of debate. In this study, we investigated the influence of BMI, together with other comorbidities, on control of asthma in a cohort of adult severe asthmatics. The following new findings are reported: (1) the optimal state of asthma control is lower in obese than in normal weight and in overweight severe asthmatics; (2) the ACQ score and the number of asthma exacerbation episodes during the last year are higher in obese than in normal weight and in overweight severe asthmatics; (3) the oral corticosteroid and LABA treatments during the previous year are higher in obese than in normal weight and in overweight severe asthmatics; (4) the type II diabetes and/or OSAS comorbidities are more frequent in obese and in overweight than in normal weight severe asthmatics. To the best of our knowledge, this is the first study investigating the role of comorbidities in a population of adult severe asthmatics with BMI as the discriminative factor. Our study adds additional information on the potential role of the high BMI in severe asthma control, which is associated with high grade of inflammation. The adipose tissue is now recognized to be a multifunctional organ with endocrine function by the production of different adipokines involved in inflammatory processes at different levels [21]. It establishes a chronic inflamed condition with increased leukocytes and cytokines that correlates with the presence of diseases associated with obesity, including type II diabetes and atherosclerosis, suggesting that obesity could be a systemic inflammatory disease. On the other hand, the term “severe asthma” is applied to patients who have refractory asthma and systemic inflammation: it remains difficult to control despite extensive reevaluation of diagnosis and management and also during an observational period of at least 6 months by an asthma specialist [10]. In children, it has been previously observed that being overweight or obese may lead to a decreased response to inhaled corticosteroids in asthma management [22]. In this study, severity indices of asthma as the poor control of asthma, ACQ test, and the number of asthma exacerbation episodes are significantly higher in obese than in normal or overweight severe asthmatics. These results may be explained with the inflammatory hypothesis that the adipose tissue generates. The obese state is characterized by the so-called low-grade systemic inflammation [21]. The white adipose tissue (WAT) of obese subjects is characterized by the increased production and secretion of a wide panel of inflammatory molecules, including cytokines, and several adipokines. Also the serum concentrations of adipokines, hormones with autocrine, or paracrine effects change in both human and murine obesity and could impact airway function leading to asthma. In addition, in the obese state, the number of macrophages infiltrating the adipose tissue increases as well as the production of IL-1*β*, leading to the activation and to the proliferation of IL-17 producing cells and to an increased airway hyperresponsiveness, a key feature of asthma [23, 24]. Asthma is a chronic inflammatory disease characterized by structural changes of the airways that may be associated with persistent symptoms and reduced lung function. The mainstay of asthma therapy is represented by inhaled corticosteroids and bronchodilator use. The aim of asthma treatment has been to minimize symptoms, optimize lung function, and prevent acute episodic deterioration (exacerbations), by lung function assessment. Recently, the new concept of “asthma control” is applied to better characterize the asthma phenotype. This is a summary term that implies a global assessment of symptoms, reliever use, lung function, and the frequency/severity of exacerbations [10]. In our study, despite the fact that the lung function parameters (FEV_1_, FVC, and FEV_1_/FVC) are similar among the studied groups, the optimal state of asthma control is completely absent in obese severe asthmatic patients, whereas it is significantly lower in overweight than in normal weight severe asthmatics, leading to the concept that, in presence of severe asthma, the patients in good nutritional status are better “controlled” in the management of the pulmonary diseases. To reinforce this message, we also found that the ACQ score and the number of asthma exacerbation episodes during the last year are significantly higher in obese severe asthmatics in comparison to the other two studied groups, leading to the concept that the severe asthmatic patient with lower BMI is better “controlled” and has a lower frequency of exacerbations in comparison with the severe asthmatic patient with higher BMI. These results are in agreement with previous studies showing that overweight patients with asthma had worse disease control as compared with normal weight patients [25] and that obesity was associated with poorer asthma control as expressed by recurrent episodes of exercise-related respiratory symptoms [26]. In addition, in our study we found that the dose of corticosteroids required during the last year and LABA treatments are significantly higher in the group of severe obese asthmatic patients, strongly supporting the concept of the refractoriness to corticosteroids in severe asthma. As a previous study assesses [10], despite intensive multidrug treatment with high-dose inhaled corticosteroids, oral corticosteroids, and LABA, many patients with severe asthma remain uncontrolled and an urgent need exists for new, more effective medications. In our sample of severe asthmatics, we demonstrate that patients with higher BMI significantly require a higher dose of drug treatment than the severe asthmatic patients with lower BMI. Our findings are strengthened by a study [27] where more than 3,000 individuals with moderate asthma treated with montelukast and beclomethasone were adversely influenced by an increased BMI, in terms of the natural history of asthma and responsiveness to asthma therapy. Also, the results of a cross-sectional study including 1,113 asthmatics are in line with our results, by reporting that obesity is associated with worse asthma outcomes, especially with an increased risk of asthma-related hospitalizations [28]. Also the comorbidities play a role in the relationship between BMI and severe asthma: it has been supposed that obesity leads to asthma not directly, but through its role in other disease processes. A recent study performed on 798 asthmatics reports that having high depressive symptoms and high BMI is associated with worse asthma control and that the relationship between BMI and worse asthma control was mediated by depressive symptoms [29]. In our study, we found that at least one of the studied comorbidities is significantly higher in obese than in normal weight or overweight severe asthmatics. This evidence is also confirmed as concerns the single association of severe asthma with the type II diabetes and with OSAS. Type II diabetes and OSAS are comorbidities for severe asthma associated with each other and with obesity [30, 31] and particularly OSAS is a complex disease entity strongly influenced by genetic factors, especially those that affect obesity and fat distribution [32]. All these results are strongly supported by previous evidence assessing that weight loss is associated with reduced number of infiltrating macrophages [33] and improves inflammatory status and the subsequent comorbidities in obesity [34], by decreasing numbers of circulating inflammatory molecules. Our results are also in agreement with a recent study reporting that OSAS, but not gastroesophageal reflux disease, may significantly contribute to poor asthma control in obese asthmatic patients [35]. In addition, recent evidences report that gastric banding provides good weight loss and significant reduction in the following comorbidities: gastroesophageal reflux disease (87%; usually immediately after surgery), asthma (81.8%), diabetes (66%), dyslipidemia (65.5%), hypertension (48%), and OSAS (33%) [36]. In obesity, type II diabetes correlates with systemic inflammatory markers, suggesting that the inflammation is functionally important. Much is already known about the relationship between obesity and type II diabetes; hence evidence exists of a relationship between asthma and type II diabetes which could produce a better understanding about the role of obesity in asthma and could result in a novel therapeutic approach to treat this susceptible population. This is the first study finding a significantly increased incidence for type II diabetes in obese and overweight severe asthmatic patients in comparison to normal weight severe asthmatic patients. The increased prevalence of type II diabetes in severe asthmatics may also be due to the adverse effects of increased concentrations of corticosteroids used for the management of the exacerbations [37]. Furthermore, we found that OSAS is significantly higher in obese severe asthmatics than in the other two studied groups. Asthma symptoms tend to be more severe during the night and asthma-related deaths are most likely to occur during the night or in the early morning. Obstructive sleep apnea syndrome, by producing sleep-disordered breathing, may contribute to asthma exacerbations and to nocturnal cough in these patients [38].

In this study the main limitation is that the BMI does not truly reflect the real individuals' fat or lean mass. Actually, the application of the bioelectrical impedance analysis could be the most appropriate tool to better examine the body composition, by the total hydration, the fat mass (FM), and the fat free mass (FFM) evaluations. In this context, further studies are needed to confirm the role of the patient's weight in the association between severe asthma and comorbidities, also by including the specific parameters of the body composition.

## 5. Conclusions

In conclusion, this study contributes to the description of severe asthma as a condition in which the body weight of the patient plays a crucial role in the asthma control and management, mainly associated with metabolic and pulmonary comorbidities.

## Figures and Tables

**Table 1:** Demographic characteristics of patients.

	Normal weight (BMI < 25)	Overweight (25.0 ≤ BMI < 30.0)	Obese (BMI ≥ 30)	*P* value
No.	42	36	24	
Body mass index (median and IQR)	22.7 (20.6–23.7)	27.5 (26.4–28.6)	32.3 (30.8–35.3)	**<**0.0001*
Age (yrs, mean ± SD)	56.6 ± 15.1	58.8 ± 12.1	53.2 ± 14.6	0.32**
Gender (males, %)	35.4	61.1	33.3	0.038***
Optimal asthma control (%)	71.4	28.6	0.0	0.023***
Mean ACQ score (median and IQR)	2.4 (1.3–3.3)	2.0 (1.1–3.3)	3.3 (3.0–3.7)	0.005*
No. of exacerbation in the last year (median and IQR)	2 (1–3)	2 (1–3)	3 (2–5)	0.052*
FEV_1_ (% of predicted, mean ± SD)	64.8 ± 16.8	63.3 ± 17.3	55.7 ± 14.9	0.09**
FVC (% of predicted, mean ± SD)	69.7 ± 15.7	68.2 ± 16.5	60.6 ± 11.6	0.06**
FEV_1_/FVC (absolute %, mean ± SD)	73.3 ± 4.4	72.5 ± 4.7	72.7 ± 9.5	0.85**
Asthma duration (yrs, median and IQR)	27.5 (21.0–37.0)	26.0 (10.0–37.5)	26.5 (19.0–40.0)	0.69*
Hospitalization for asthma ever (%)	81.0	8.33	75.0	0.72***
Intensive Care Unit for asthma ever (%)	28.6	27.8	33.3	0.89***
OCS in the previous year for asthma (%)	50.0	72.2	79.2	0.029***
LABA use in the previous year (%)	69.0	83.3	95.8	0.027***
Aspirine intolerance (%)	14.3	13.9	4.2	0.42***
Antidepressant use (%)	7.1	8.3	4.2	0.82***
Antisthaminic use (%)	64.3	52.8	75.0	0.21***

IQR: interquartile range; ACQ: Asthma Control Questionnaire, Juniper scores; OCS: oral corticosteroids; LABA: long-acting *β*_2_-agonists.

Statistical significance among groups was evaluated by *Kruskall-Wallis test, **analysis of variance, and ***χ ^2^ test.

**Table 2:** Trigger precipitating factors and inflammatory markers.

	Normal weight (BMI < 25)	Overweight (25.0 ≤ BMI < 30.0)	Obese (BMI ≥ 30)	*P* value
Allergic sensitization (%)	63	66	86	0.16*
Total IgE (UI/mL, median and IQR range)	282.5 (87.5–684.0)	188.0 (102.3–836.3)	202.0 (36.3–326.0)	0.17**
Blood eosinophils/mm^3^ (median and IQR range)	248 (145–379)	245 (122–756)	192 (154–310)	0.49**

Allergic sensitization was evaluated as at least one positive skin prick test; IQR: interquartile range.

Statistical significance among groups was evaluated by *χ ^2^ test and **Kruskall-Wallis test.

**Table 3:** Comorbidities associated with severe asthma.

	Normal weight (BMI < 25)	Overweight (25.0 ≤ BMI < 30.0)	Obese (BMI ≥ 30)	*P* value
At least one comorbidity (%)	61.5	74.3	91.7	**0.031**
Type II diabetes (%)	0	8.3	37.5	**<0.0001**
OSAS (%)	0	2.8	16.7	**0.0081**
Gastroesophageal reflux (%)	33.3	50.0	52.6	0.24
Systemic hypertension (%)	11.9	25.0	33.3	0.10
Left ventricle failure (%)	4.8	16.7	12.5	0.23
Osteoporosis (%)	21.4	19.4	29.2	0.66
Bronchiectasis (%)	2.4	2.8	8.3	0.44
Tuberculosis (in the past) (%)	4.8	0	4.2	0.43
Psychologic factors [anxia/depression] (%)	7.1	8.3	4.2	0.82

OSAS: obstructive sleep apnea syndrome.

Statistical significance among groups was evaluated by *χ* ^2^ test.

## Abbreviations

BMI: Body mass index
OSAS: Obstructive sleep apnea syndrome
COPD: Chronic obstructive pulmonary disease
ACQ: Asthma control questionnaire
GINA: The global initiative for asthma
FEV_1_: Forced expiratory volume in the 1st second
FVC: Forced vital capacity
SD: Standard deviation
LABA: Long acting beta2-agonists
WAT: White adipose tissue
FM: Fat mass
FFM: Fat free mass
IQR: Interquartile range
OCS: Oral corticosteroids.
